# Low prevalence of clinical decision support to calculate caloric and fluid intake for infants in the neonatal intensive care unit

**DOI:** 10.1038/s41372-019-0546-z

**Published:** 2019-12-09

**Authors:** Gustave H. Falciglia, Karna Murthy, Jane L. Holl, Hannah L. Palac, Donna M. Woods, Daniel T. Robinson

**Affiliations:** 1 Department of Pediatrics, Northwestern University, Feinberg School of Medicine, Chicago, IL USA; 2 Ann & Robert H. Lurie Children’s Hospital of Chicago, Chicago, USA; 3 Children’s Hospital Neonatal Consortium, Kansas City, MO USA; 4 Center for Health Services & Outcomes Research, Northwestern University, Feinberg School of Medicine, Chicago, IL USA; 5 nPhase, Inc, Encinitas, CA USA

**Keywords:** Paediatrics, Health services

## Abstract

**Background:**

Clinical decision support (CDS) improves nutrition delivery for infants in the neonatal intensive care unit (NICU), however, the prevalence of CDS to support nutrition is unknown.

**Methods:**

Online surveys, with telephone and email validation of responses, were administered to NICU clinicians in the Children’s Hospital Neonatal Consortium (CHNC). We determined and compared the availability of CDS to calculate calories and fluid received in the prior 24 h, stratified by enteral and parenteral intake, using McNemar’s test.

**Results:**

Clinicians at all 34 CHNC hospitals responded with 98 of 108 (91%) surveys completed. NICUs have considerably less CDS to calculate enteral calories received than enteral fluid received (32% vs. 82%, *p* < 0.001) and less CDS to calculate parenteral calories received than parenteral fluid received (29% vs. 82%, *p* < 0.001).

**Discussion:**

Most CHNC NICUs are unable to reliably and consistently monitor caloric intake delivered to critically ill infants at risk for growth failure.

## Background

Clinical decision support (CDS) is a type of information technology that provides timely and patient-specific information to clinicians [1, 2]. CDS improves patient outcomes when provided at the time of decision making and when integrated into clinicians’ workflow [3, 4]. CDS provides real-time support to clinicians during order entry, by improving the accuracy of complicated calculations [5]. For example, CDS has been shown to generate the volume of fluid intake needed to resuscitate pediatric burn patients faster and with less error compared to manual calculations with pen and paper [6]. Furthermore, antibiotics with narrow therapeutic windows are dosed more accurately with CDS [7].

CDS has also been shown to improve the quality and safety of nutrition of preterm infants in the neonatal intensive care unit (NICU) [8–10]. Enhanced nutrition, including the delivery of appropriate calories and macronutrients, is associated with improved outcomes including better growth and decreased risk of neurodevelopmental impairment in preterm infants [11, 12]. Nevertheless, half of preterm infants in North America are discharged home from the NICU at less than the tenth percentile for weight [13]. Deficits in caloric intake accrue in the first weeks of life in preterm infants [14, 15], while a decline in protein intake occurs during the transition from parenteral fluids to enteral nutrition [16–18]. Using CDS in the NICU to assist with ordering parenteral nutrition has been shown to reduce error and improve protein intake and growth; [8–10] however, studies to date are from single centers. No study has determined the prevalence of CDS in NICUs to support clinical decisions or to monitor the quality of nutrition delivery.

The aim of this study is to report the prevalence of CDS to calculate nutrition and fluid intake at 34 regional NICUs, affiliated with the Children’s Hospitals Neonatal Consortium (CHNC) [19]. The study describes the availability of CDS to calculate nutrition and fluids received in the prior 24 h and to estimate projected nutrition and fluids that an infant should receive in the subsequent 24 h, based on the orders placed in the electronic health record (EHR). We hypothesize that (1) few NICUs have CDS to calculate intake despite universal implementation of EHR and computer order entry and (2) more NICUs have CDS that calculates fluid intake compared with caloric or macronutrient intake.

## Methods

### Cohort and data collection overview

The study was approved by the Institutional Review Board of the Ann & Robert H. Lurie Children’s Hospital of Chicago. Electronic surveys were sent via Typeform (Barcelona, Spain) to the physician leader at each NICU affiliated with the CHNC in the spring of 2017 (survey link) [19]. The CHNC is a group of regional, level IV NICUs from children’s hospitals in the United States and Canada [20]. To improve the validity of the study, surveys were also sent to 2–3 additional clinicians at each NICU. Additional respondents from each NICU were recommended by the physician leaders, based on their interest in nutrition, and included at least one registered dietitian. Responses, including any discrepancies, were further reviewed with at least one respondent from each site by study investigators (GF and KM). Finally, in the fall of 2018, respondents were asked to identify any recent changes since the initial survey. Additional details regarding the survey are reported in the Supplementary Table in accordance with the “Checklist for Reporting Results of Internet E-Surveys (CHERRIES)” [21].

### Survey questions

Respondents were asked about the EHR system in their NICU and time since implementation. Time since implementation of the current EHR was categorized as 0–3, 4–6, 7–9, ≥10 years or unknown. Respondent roles were self-reported or determined using each hospital’s online directory.

Respondents were asked about the methods used in their NICU to calculate received caloric and fluid intake, in kcal/kg and mL/kg, respectively. Similar questions were asked about projected caloric and fluid intake. The survey provided relevant examples and images to clarify the differences. The response options for the methods of calculation were determined from pilot data and included: (a) manual calculation, using EHR data hand copied with pen and paper (i.e., no CDS); (b) typed calculation, using EHR data re-typed into a CDS software; (c) automated calculation, performed by CDS without any additional hand copying or retyping of data; and (d) other. If the respondents selected typed, automated, or other, they were asked whether the method had additional capacity to calculate individual macronutrients in g/kg. They were also asked whether the resultant calculations for calories and fluid included enteral (i.e., human milk or formula) and parenteral nutrition (i.e., intravenous solutions with dextrose and protein as well as lipids).

### Response review

Discrepancies between respondents regarding the method used for calculating nutrition and fluid intake were reviewed with at least one respondent from each NICU by telephone or email. Though not requested, respondents frequently included screen shots of the CDS to clarify discrepancies (with patient health information removed). We did not consider any tool that only calculates total calories, total grams of macronutrients, or total milliliters of fluid as CDS because clinicians are required to manually divide total values by weight to generate estimates of kcal/kg, g/kg, and mL/kg. We report all CDS, independent of frequency of use or profession of user.

The reviews further clarified whether different CDS were used to calculate caloric and fluid intake from enteral and parenteral sources. To ensure that the existence of a CDS was not overlooked, respondents were also asked about other commonly used types of CDS. For example, respondents were routinely asked about summary reports that often accompany a parenteral nutrition order screen. These reports generate projected parenteral calories and fluid from clinician orders, using the ordered delivery rate.

During the review, additional details regarding the CDS were systematically elicited and included: (1) whether the CDS was integrated within the EHR or standalone (operating separately from the EHR) [22], and (2) whether fluid received was calculated, using the daily weight (defined as the actual, measured weight for the day) versus a dosing weight (defined as a weight used for medications after the first week of life and typically updated weekly). This detail was elicited after noting that several respondents reported manually calculating fluid received despite having an automated CDS for the calculation. These respondents indicated a preference for calculating fluid received using a weight different from the weight used in the automated CDS calculation. Finally, each respondent was provided with a summary of their NICU’s CDS and given the opportunity to provide corrections.

### Analyses

The distribution of respondents’ roles was described and included physician, dietitian, nurse practitioner or physician assistant, and pharmacist. Type and implementation time of the EHR (the oldest recalled time reported was chosen for each NICU) were described.

The presence or absence of CDS at each NICU was determined for calories and fluid received, stratified by enteral and parenteral delivery. The same analysis was performed for projected calories and fluid. The outcome, any CDS, was defined as present if a NICU relied on any non-manual method to calculate specific values. CDS was further characterized as “typed” or “automated” and “integrated” or “standalone”. Finally, the presence of any CDS to calculate calories and fluid was compared using the exact McNemar’s test for correlated proportions [23]. Statistical significance was defined by a two-tailed test and *p* < 0.05 for all testing using Stata 15.0 (College Station, TX, USA).

## Results

### Survey response and NICU characteristics

One hundred eight (108) surveys were sent to clinicians at the 34 NICUs participating in CHNC with 98 unique responses (91%), including at least two responses from each NICU (median response per NICU: 3; IQR: 3, 3). Most respondents were physicians (57%); respondents also included dietitians (34%), nurse practitioners or physician assistants (7%), and pharmacists (2%). CHNC NICUs are using Epic [18/34 (53%)], Cerner [14/34 (41%)], Allscripts [1/34 (3%)], and Meditech [1/34 (3%)] as their EHR; 22 (65%) were implemented at least 6 years ago and four hospitals implemented a new EHR during the study period.

### CDS to calculate nutrition and fluid received

**Fig. 1:**
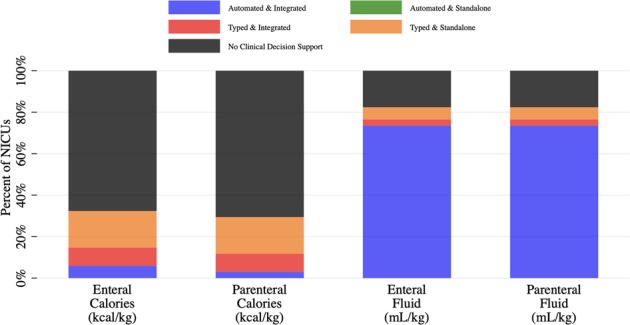
Clinical decision support for intake received in prior 24 h

Most NICUs lack CDS to calculate enteral or parenteral calories received in kcal/kg (Fig. 1). NICUs without CDS relied on data transcribed by hand from the EHR to a hard-copy data collection sheet for calorie and macronutrient calculations.

The majority of NICUs with CDS to calculate enteral and parenteral calories received do not have an automated CDS. Clinicians must retype data from the EHR into a calculator within the same EHR or into a calculator separate from the EHR. Only one surveyed NICU has an automated CDS that calculates both enteral and parenteral calories received. Another NICU has an automated CDS that calculates enteral calories received within a progress note through an automatic update of EHR text. None of the remaining NICUs with the same type of EHR reported having this automated CDS. Enteral protein, fat and carbohydrates received are calculated in 73%, 45 and 36% of NICUs with CDS to calculate enteral calories received. Parenteral protein, fat and carbohydrates received are calculated in 90%, 60 and 50% of NICUs with CDS to calculate parenteral calories received.

For both enteral (Table 1a) and parenteral intake (Table 1b), participating NICUs are more likely to have CDS to calculate fluid received than calories received. Most CDS for enteral and parenteral fluid received are automated and integrated within the EHR (Fig. 1). These CDS summarize fluid intake from all sources, divide it by the weight and display the results in an intake and output report. The weight used in the automated CDS calculation of fluid received varied with 60% of NICUs reportedly using the daily weight and 40% using the dosing weight. Two NICUs had a typed CDS tool to calculate enteral and parenteral fluid received. The remaining NICUs, without CDS to calculate enteral and parenteral fluid received, used an EHR summary of total fluid intake that required clinicians to manually divide by weight to obtain fluid received in mL/kg.

**Table 1:** Comparison of clinical decision support (CDS) for intake received in prior 24 h (each cell represents the number of NICUs)

### CDS to calculate projected nutrition and fluid

**Fig. 2:**
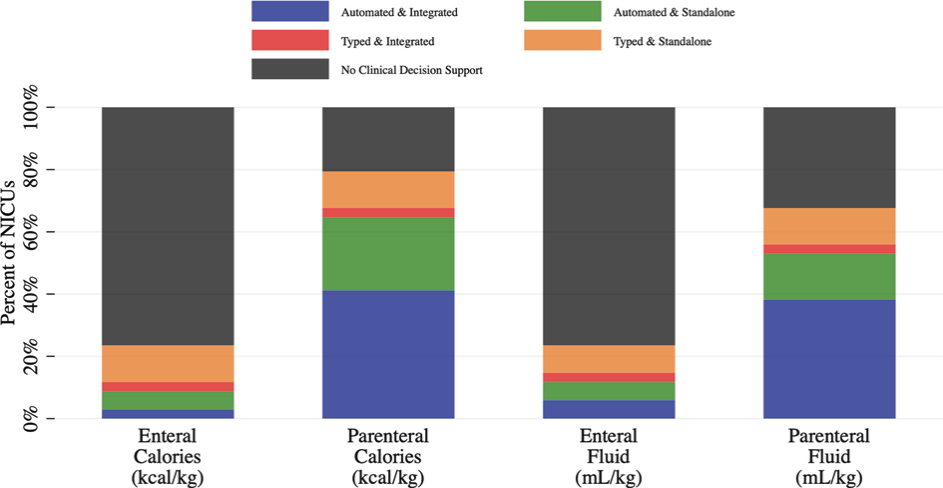
Clinical Decision Support for Projected Intake in Subsequent 24 h

While few NICUs have CDS to calculate projected enteral calories, most have CDS to calculate projected parenteral calories (Fig. 2). Automated CDS that generate projected parenteral calories are found in summary reports alongside parenteral nutrition order screens. NICUs using typed CDS (i.e., order data are retyped into a standalone software outside the EHR) are generally able to calculate both enteral and parenteral projected calories. Projected enteral protein, fat and carbohydrates are calculated in 88%, 88 and 63% of NICUs with CDS to calculate projected enteral calories. Projected parenteral protein, fat and carbohydrates are calculated in 89%, 70 and 74% of NICUs with CDS to calculate projected parenteral calories; however, many of these values are directly set by the clinician during the ordering process. For both enteral (Table 2a) and parenteral intake (Table 2b), there were no differences in the prevalence of CDS to calculate projected calories compared with projected fluid.

**Table 2:** Comparison of clinical decision support (CDS) for projected intake in subsequent 24 h (each cell represents the number of NICUs)

## Discussion

In 2018, despite universal EHR implementation, most regional level IV NICUs in the CHNC still rely on the manual calculation of nutrition intake by clinicians. Few NICUs have the capacity to provide clinicians with readily available feedback of data to facilitate the delivery of nutrition to critically ill infants [24]. This lack of CDS impedes the healthcare systems’ ability to monitor the quality of care, an important benefit of automated data collection systems. Most participating NICUs cannot monitor whether cumulative, ordered nutrition is at recommended levels, nor monitor the gap between the nutrition projected by clinician’s orders and the nutrition actually received by infants. It is difficult to improve what is not measured [24, 25].

Although EHR implementation necessitates a large financial investment [26, 27], the expense is justified by the EHR’s ability to provide thorough patient information efficiently [28]. Yet only one of the 34 NICUs reported CDS that automatically calculates enteral and parenteral nutrition intake. Several NICUs reporting typed CDS require clinicians to redundantly retype data from one flowsheet of the EHR into another flowsheet within that same EHR. Furthermore, an innovative CDS (e.g., automatic population of enteral calories received within a progress note) has not been replicated among NICUs in different healthcare systems that use the same EHR.

The calculations needed to accurately assess various aspects of nutrition can be performed manually; however, manual calculation contains an inherent risk of error while computers (i.e., CDS) excel at performing calculations accurately, rapidly, and repeatedly [6, 7]. In the aforementioned example of pediatric burn patients [6], manual calculation of the volume of fluid intake needed to resuscitate patients resulted in small errors (>25% of correct value) 51% of the time and large errors (>100% of correct value) 21% of the time. A greater cause for concern, however, is that the lack of any CDS and, in particular of any automated CDS, may result in the calculations simply not being performed. As a result, clinicians may only focus on available data or data that are easier to calculate, such as fluids [15]. We believe that discussion of both fluid and caloric intake is equally important.

Assuming that recopying or retyping data takes 2–3 min to generate a value for nutrition intake, a clinician with ten patients would require an extra 30 min just to acquire data and perform calculations. This is neither efficient nor reliable. Rather, clinicians should spend their time and cognitive skills determining optimal nutrition for growth while balancing competing issues such as cardiopulmonary status and the risk-benefit assessment of a central line [18, 29–31]. The transition from parenteral to exclusive enteral nutrition requires clinicians to decide how best to wean parenteral nutrition, whether to maintain central line access, and when to fortify enteral feeds [16, 32, 33]. Balancing these tradeoffs is complicated and may require multiple revisions to nutrition orders which likely impact projected nutrition intake.

Ideally, clinicians should receive immediate and accurate estimates of projected nutrition intake with each revised prescription so that they may balance achieving ideal nutrition delivery with other potential tradeoffs. Although many NICUs have CDS to calculate projected parenteral caloric and fluid intake, few NICUs have CDS to calculate projected enteral caloric or fluid intake (Table 2). This suggests that calculating aspects of enteral nutrition may require advanced logic that is currently summarized via free-text comments, rather than discretely within the order (e.g., “start feeds at 5 mL every 3 h and increase by 5 mL every feed until goal feeds”).

There are limitations to this study. The survey used a convenience sample of clinicians and this may have introduced unknown biases. Furthermore, discrepancies between respondents existed because clinicians were unaware of CDS, aware of CDS but reported the continued manual calculation of caloric or fluid intake, or were unclear about the difference between received and projected intake. We attempted to mitigate these potential biases and discrepancies by duplicating the surveys, seeking out clinicians with an interest in nutrition, and clarifying discrepancies with respondents via telephone or email correspondence. We specifically asked about common CDS to mitigate underreporting and we sent summaries of each NICU’s findings to the respondents for correction. Finally, the study captured current CDS at regional, level IV NICUs across the United States and Canada which may not be applicable to NICUs in general hospitals. Thus, our study may have overestimated the prevalence of CDS since there are more NICUs housed in hospital systems that are not exclusively dedicated to pediatric patients and that must distribute healthcare resources to a broader population.

In conclusion, the prevalence of CDS to calculate nutrition intake in North American NICUs is very low. Quality healthcare for critically ill infants requires the delivery of adequate nutrition to support growth and development [34]. Developing CDS that provides comprehensive data on nutrition management will require investments by health care institutions and would benefit from standardization of measurements, including the source of weight used for calculations. Advances can be expected to support the delivery of high quality nutrition to any critically ill population.

## Supplementary information

Supplemental Table
