# Supplementary material for: Low prevalence of clinical decision support to calculate caloric and fluid intake for infants in the neonatal intensive care unit
Source: J Perinatol. 2019 Dec 9;40(3):497–503. doi: 10.1038/s41372-019-0546-z (PMC7042157; doi:10.1038/s41372-019-0546-z)
Supplement: Supplementary file 1 — Supplemental Table [file 41372_2019_546_MOESM1_ESM.docx]

*Supplemental Table: Checklist for Reporting Results of Internet E-Surveys (CHERRIES)*

| **Item Category** | **Checklists Item** | **Explanation** |
| --- | --- | --- |
| Design | Describe survey design | Convenience sample of neonatal intensive care unit (NICU) clinicians affiliated with the Children’s Hospital Neonatal Consortium (CHNC) (see “Methods”, paragraph 1) |
| Institutional Review Board (IRB) Approval & Informed consent process | IRB approval | Study was approved by the Ann & Robert H. Lurie Children’s Hospital IRB (see “Methods”, paragraph 1) |
|  | Informed consent | The study description using language approved by the IRB was included in the recruitment email. The description included:   - Identification of primary investigator - Purpose and estimated length of survey - Survey was optional and voluntary - Only completed surveys will be collected - Possibility of follow up after survey - Identity of respondents obtained through email follow-up (i.e., email address and name) would be kept securely   Respondents began the survey by indicating that they voluntarily agreed to participate in the research study |
|  | Data protection | No personal information of respondents was collected through survey. Respondent identifiers included with follow-up (i.e. email addresses) were kept securely on a hospital encrypted computer. |
| Development and Pre-testing | Development and testing | Authors (GF, KM, JH, DW, DR) developed survey questions through an iterative approach. Prior to sending survey, the survey was tested on Typeform\| to ensure usability (GF). |
| Recruitment process & description of sample having access to the questionnaire | Open versus closed survey | Closed - only physician leaders at each NICU or those recommended were given the link to the survey. Survey completion was monitored by the primary author (GF). |
|  | Contact mode | Initial contact was via email to physician leaders at CHNC. Physician leaders then recommended clinicians (including non-physicians) with an interest or knowledge of the subject. |
|  | Advertising the survey | Survey was not advertised online. |
| Survey administration | Web vs. email | Surveys were created through Typeform\| (<http://www.typeform.com>; Barcelona, Spain) and sent via email. |
|  | Context of website | Not applicable. Surveys were sent directly to respondents and they were not accessed through an initial website. |
|  | Mandatory vs. voluntary | Voluntary |
|  | Incentives | Respondents who completed a survey were given a $10 Starbucks electronic gift card |
|  | Time/date | Initial surveys were sent to physician leaders at each CHNC NICU in the spring of 2017. Additional surveys were sent to recommended clinicians at each site in late 2017 and early 2018. Clarification of responses occurred throughout this period. Follow-up to determine if responses changed occurred in fall of 2018. See “Methods”, paragraph 1. |
|  | Randomization of items or questions | Items were not randomized |
|  | Adaptive questioning | If respondents reported a non-manual method to calculate received or projected calories, then they were asked 1) whether this method calculated macronutrient intake, and 2) what type of intake (enteral or parenteral) was included in the calculation. |
|  | Number of items | There were a maximum of 29 questions and minimum of 21 questions depending on responses (see above, “adaptive questioning”) |
|  | Number of screens (pages) | Typeform displayed one question at a time and questions were not grouped by pages. |
|  | Completeness check | Survey could not be submitted unless all questions were answered |
|  | Review step | Respondents were free to go back and edit answers prior to submitting completed survey. |
| Response Rates | Unique site visitors | Not applicable. The survey was not accessed by the respondent from a website. Rather, the survey link was sent directly via email to potential respondents. |
|  | View rate (ratio of unique survey visitors / unique site visitors) | Not applicable. |
|  | Participation rate (ratio of those who agreed to participate / who were sent survey) | There were 108 surveys sent via email   - 98 clinicians agreed to complete the survey and completed the survey, - 4 clinicians agreed to complete the survey and did not complete the survey, - 3 clinicians declined to complete the survey, and - 3 clinicians did not reply.   Therefore, the participation rate was 102/108 (94%) |
|  | Completion rate (ratio of users who finished the survey / users who agreed to participate) | The completion rate was 98/102 (96%) of users who agreed to complete survey. In summary, 98/108 (91%) surveys sent were completed. See “Results”, paragraph 1. |
| Preventing multiple entries from the same individual | Cookies used | Cookies were not used by Typeform to assign a unique user identifier to each client computer. It was possible for a respondent to complete the survey more than once; however, responses were monitored as the surveys were gradually completed. Follow-up emails were promptly sent to respondents to confirm survey completion, clarify responses and send the electronic gift card (see above). No respondent answered the survey more than once. |
